# Pilot GWAS of caries in African-Americans shows genetic heterogeneity

**DOI:** 10.1186/s12903-019-0904-4

**Published:** 2019-09-18

**Authors:** E. Orlova, J. C. Carlson, M. K. Lee, E. Feingold, D. W. McNeil, R. J. Crout, R. J. Weyant, M. L. Marazita, J. R. Shaffer

**Affiliations:** 1 Department of Human Genetics, Pittsburgh, USA; 2 Department of Biostatistics, Graduate School of Public Health, Pittsburgh, USA; 3 Center for Craniofacial and Dental Genetics, Dept. of Oral Biology, School of Dental Medicine, University of Pittsburgh, Pittsburgh, PA USA; 4 Departments of Psychology, & Dental Practice and Rural Health, West Virginia University, Morgantown, USA; 5 Department of Periodontics, School of Dentistry, West Virginia University, Morgantown, WV USA; 6 Department of Dental Public Health and Information Management, Pittsburgh, USA; 7 Clinical and Translational Sciences Institute, University of Pittsburgh, Pittsburgh, PA USA; 8 Department of Psychiatry, School of Medicine, University of Pittsburgh, Pittsburgh, PA USA

**Keywords:** Child, Adult, Genetic predisposition to disease, Humans, Dentistry, Public health, Healthcare disparities*

## Abstract

**Background:**

Dental caries is the most common chronic disease in the US and disproportionately affects racial/ethnic minorities. Caries is heritable, and though genetic heterogeneity exists between ancestries for a substantial portion of loci associated with complex disease, a genome-wide association study (GWAS) of caries specifically in African Americans has not been performed previously.

**Methods:**

We performed exploratory GWAS of dental caries in 109 African American adults (age > 18) and 96 children (age 3–12) from the Center for Oral Health Research in Appalachia (COHRA1 cohort). Caries phenotypes (DMFS, DMFT, dft, and dfs indices) assessed by dental exams were tested for association with 5 million genotyped or imputed single nucleotide polymorphisms (SNPs), separately in the two age groups. The GWAS was performed using linear regression with adjustment for age, sex, and two principal components of ancestry. A maximum of 1 million adaptive permutations were run to determine empirical significance.

**Results:**

No loci met the threshold for genome-wide significance, though some of the strongest signals were near genes previously implicated in caries such as antimicrobial peptide *DEFB1* (rs2515501; *p* = 4.54 × 10^− 6^) and *TUFT1* (rs11805632; *p* = 5.15 × 10^− 6^). Effect estimates of lead SNPs at suggestive loci were compared between African Americans and Caucasians (adults *N* = 918; children *N* = 983). Significant (*p* < 5 × 10^− 8^) genetic heterogeneity for caries risk was found between racial groups for 50% of the suggestive loci in children, and 12–18% of the suggestive loci in adults.

**Conclusions:**

The genetic heterogeneity results suggest that there may be differences in the contributions of genetic variants to caries across racial groups, and highlight the critical need for the inclusion of minorities in subsequent and larger genetic studies of caries in order to meet the goals of precision medicine and to reduce oral health disparities.

## Background

Dental caries is a complex disease influenced by genetic and environmental factors, including diet, oral hygiene, oral bacteria such as *Streptococcus mutan*s, tooth morphology and placement, the composition and flow rate of saliva, fluoride exposure, and access to oral health care [1–4]. Genetic determinants of caries differ, in part, based on tooth surface and tooth type (primary versus permanent) [5, 6]. Etiological mechanisms can additionally involve gene-by-sex and gene-by-environment interactions [7, 8].

According to the National Health and Nutrition Examination Survey (NHANES), caries affects the majority of children (i.e., 23% by age 5 years, 56% by age 8, 67% by age 19), and adults (91%) and is the most common chronic disease in the United States [9–11]. Lack of treatment leads to serious co-morbidities that greatly impair quality of life [9].

Although caries has declined in the United States since the mid-twentieth century, the caries rate in young children has increased in recent years, and disparities persist between racial/ethnic, demographic, and socioeconomic groups [10–12]. Caries prevalence in primary teeth is 42% higher in non-Hispanic black children compared with non-Hispanic Caucasian children. Non-Hispanic black children have double the rate of untreated tooth decay in primary teeth compared to non-Hispanic Caucasian children [11], and among adults, non-Hispanic blacks have nearly double the rate of untreated decayed teeth (42%) of non-Hispanic Caucasians (22%) [10].

Some disparity is explained by sociocultural differences between racial groups. African Americans are less likely to have access to and utilize oral health care [13, 14]. Other factors include differences in caretaker fatalism and oral health education [15], socioeconomic status, and transmission of cariogenic bacteria [16]. Genetic differences in caries predisposition are known: the 2% of African American children with localized juvenile periodontitis – a disease more common in African Americans – have fewer carious teeth than others, likely due to a variant in the gene encoding a protective component of saliva [17]. Other differences include those in immunity genes and propensity toward cariogenic oral flora [18]. While inter-racial genetic differences influence dental features [19], there is a dearth of studies on the role of genetics in differences in dentition across racial and ethnic groups.

Although dental caries is estimated to be 30–50% heritable [1, 5, 6, 20], few specific caries-related genes have been discovered, with the majority of these identified in Caucasians [21]. Yet, it is known that some complex diseases exhibit differences in their predominant genetic architecture across races [22–24]. Genetic markers for disease vary in frequency between races, and the effect sizes of the genetic variants can display large heterogeneity [25]. Indeed, up to 25% of GWAS tagSNPs show effect heterogeneity by ancestry [26]. Thus it is possible that there are different genetic risk factors for caries operating between races, or that the effects of risk variants are dissimilar. In spite of this, adequate information is lacking regarding the disease process in vulnerable groups such as racial/ethnic minorities; in particular, few studies have focused on the oral health of African Americans [12]. Genome-wide association studies (GWAS) of dental caries in African American samples have not been performed, and although African-Americans are a large US minority group, little work has been done to understand their dental genetics. In this study, we describe a pilot caries GWAS in African American children and adults to generate hypotheses about the genetics of dental caries in African Americans. We consider primary and permanent dentition separately because previously work has estimated that only 18% of covariation in primary vs permanent tooth caries is due to common genetic factors [6]. Furthermore, we compare the GWAS scans in African Americans to analogous analyses in Caucasian children and adults to determine whether there is heterogeneity present between the two racial groups.

## Methods

### Study sample

One hundred nine African American adults (aged > 18 years) and 96 African American children (3–12 years) were recruited through the Center for Oral Health Research in Appalachia (COHRA, cohort COHRA1), a joint study of the University of Pittsburgh and West Virginia University [27]. Briefly, all participants provided consent or assent with written parental informed consent, in accordance with the Institutional Review Board policies of the University of Pittsburgh and West Virginia University. Two clinical examination sites were located in Pennsylvania and four in West Virginia. Admixed African ancestry was verified using Principal Component Analysis (PCA) with respect to HapMap controls from Europe, Asia, Africa, and Central/South America. Participants were genotyped for approximately 550,000 single nucleotide polymorphisms (SNPs) using the Illumina Human610-Quad Beadchip (Illumina, Inc., San Diego, CA). Genetic data were rigorously cleaned and quality-checked as previously described [28], and imputed to the 1000 Genomes Project (June 2011) phase 1 reference panel using SHAPEIT (for pre-phasing) [29] and IMPUTE2 [30]. SNPs were filtered for INFO score > 0.5, and MAF > 5% (separately for each age group). SNPs were not filtered for HWE due to the admixed nature of the African American population. Quality filters included participant call rates > 90% and SNP call rates > 99%. Approximately 4.9 million SNPs passed quality control and were included in the GWASs. Identical analyses were performed in COHRA-recruited cohorts of 918 Caucasian adults and 983 children (results for these cohorts have been previously published) [28, 31]. The same filters were used in Caucasians (separately for each age group) along with a filter for HWE (*p*-value > 10^− 4^). STROBE guidelines were followed for this observational study.

### Quantitative caries phenotypes

Ascertainment of caries status was conducted with a dental explorer by either a licensed dentist or a dental hygienist. The assessments were done in exam rooms with a dental chair and dental examination light on dried teeth, and were mutually calibrated at the start of the study and several times over the course of data collection via a review of data collection techniques followed by reliability testing [27]. Inter- and intra-rater reliability of caries assessments was high [27]. From these assessments, the following caries phenotypes were generated: the DMFS index (**D**ecayed, **M**issing, and **F**illed Tooth **S**urfaces) and DMFT index (**D**ecayed, **M**issing, and **F**illed **T**eeth) in adults, and the dfs index (**d**ecayed and **f**illed deciduous tooth **s**urfaces) and dft index (**d**ecayed, and **f**illed deciduous **t**eeth) in children. These caries indices represent the count of affected tooth surfaces or teeth, in accordance with the World Health Organization DMFS/dfs or DMFT/dft scales [32] and established dental caries research protocols [33, 34]. For 31 of the 96 children in the African American pediatric cohort with mixed dentition, and 378 of 983 children in the Caucasian pediatric cohort with mixed dentition, both DMFS/DMFT and dfs/dft indices were scored at the time of the assessment. For the purposes of this study only dfs/dft measures were tested for association in the pediatric cohorts. White spots were included in the DMFS/DMFT and dfs/dft counts because their inclusion has been shown to increase caries heritability estimates and thus improve power to detect association in gene mapping [6].

### Statistical model

The GWASs were performed separately in adults (for DMFT and DMFS) and children (for dft and dfs) using linear regression while adjusting for age, sex, and two principal components of ancestry in PLINK v1.9 [35]. Statistical significance was determined using adaptive imputation with a maximum number of 1,000,000 permutations per SNP as implemented in PLINK. *P*-value thresholds incorporated the burden of multiple testing: genome-wide significance was defined as *p*-value less than 5 × 10^− 8^ and suggestive significance as p-value less than 5 × 10^− 6^. Results were visualized in Manhattan plots using R (v3.2.0) [36].

### Results annotation and comparison with Caucasian caries GWASs

Genes within 500 kb of the top associated SNP in each locus were queried for corroborating biological connections to dental caries in public databases, including OMIM, PubMed, and ClinVar. In addition, GREAT [37] was used to assess the functions of cis-regulatory regions of the associated loci using default parameters.

Heterogeneity in effect sizes between the GWAS results of African Americans and Caucasians were compared via Cochran’s Q statistic. The effect sizes for the lead SNPs at suggestive (*p*-value ≤5 × 10^− 6^) loci observed in African Americans were compared with the effect sizes of the same SNPs in Caucasians, if present. Not all suggestively-associated lead SNPs in African Americans were tested for heterogeneity because MAF and quality controls filters yielded different sets of SNPs retained for African Americans and Caucasians. Specifically, the numbers of loci tested for heterogeneity were 17 of 25 for DMFT, 11 of 12 for DMFS, 20 of 26 for dft, and 12 of 18 for dfs. The genome-wide significance threshold for heterogeneity tests was *p*-value ≤5 × 10^− 8^.

## Results

**Fig. 1:**
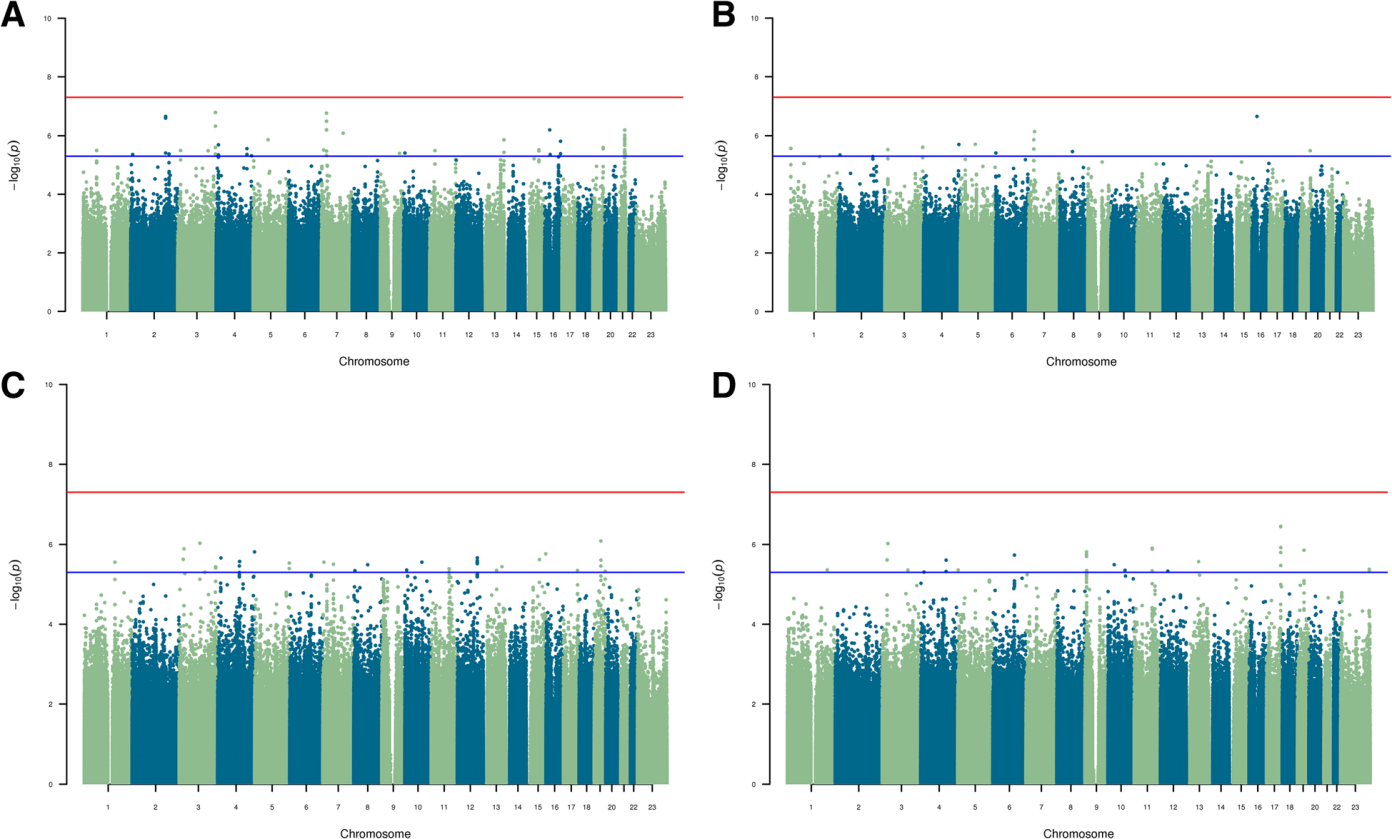
Manhattan plots for the permuted results of **a** permanent DMFT **b** permanent DMFS **c** primary dft, and **d** primary dfs GWASs. *P*-values are log_10_-transformed. The red line signifies genome-wide significance (*p*-value ≤5 × 10^− 8^), and the blue line signifies suggestive significance (*p*-value < 5 × 10^− 6^)

Four GWASs of indices of dental caries were performed: DMFS and DMFT in 109 African American adults, and dfs and dft in 96 African American children. Cohort demographics are shown in Table 1. The GWAS in African Americans did not yield associations at genome-wide significance (*p*-value ≤5 × 10^− 8^) for any phenotype (Fig. 1), while several loci with potential roles in caries etiology were associated at suggestive significance (p-value ≤5 × 10^− 6^).

**Table 1:** Demographics of African-American and Caucasian cohorts included in the study

Race	African American		Caucasian	
Cohort	Adults	Children	Adults	Children
N	109	96	918	983
Age; mean (range)	29.15 (18–58)	7.30 (3–11)	33.96 (18–64)	6.37 (3–11)
Male (%)	38 (34.9%)	48 (50.0%)	683 (32.8%)	616 (50.7%)
Female (%)	71 (65.1%)	48 (50.0%)	910 (57.1%)	599 (49.3%)
DMFT/dft; mean (range)	7.17 (0–28)	2.21 (0–12)	10.39 (0–28)	1.96 (0–17)
DMFS/dfs; mean (range)	18.2 (0–106)	4.90 (0–35)	23.00 (0–122)	3.85 (0–53)
PCs	2	2	2	2
Genotyped SNPs	529,015	529,837	526,525	510,212
Imputed SNPs	4,907,119	4,912,366	4,915,678	4,931,991

*PCs* number of principal components adjust for in the GWAS

### GWASs of caries in the permanent dentition in African Americans

The GWAS of DMFT yielded 94 suggestive (p-value ≤5 × 10^−6^) SNPs across 25 distinct loci. The GWAS of DMFS yielded 23 suggestive SNPs across 11 distinct loci. These loci and corroborating evidence for nearby genes are listed in Table 2 (DMFT) and Table 3 (DMFS). Many of the top loci for the two phenotypes overlapped (rs6947348, rs12171500, chr3:194035416, rs12488352, rs1003652). GREAT regulatory analysis results are available in the **Appendix**.

**Table 2:** Suggestive loci observed for DMFT

Lead SNP	CHR	BP	African-American cohort			Caucasian cohort		Q Statistic P-value	Nearby Gene(s) and Corroborating Evidence
			Effect Size	SE	GWAS *P*-value	Effect Size	SE		
rs74086974	1	71,335,753	4.522	0.904	3.18E-06				***PTGER3*** Candidate gene for role in outcome of periodontal therapy and preterm birth [38]. ***ZRANB2*** Affects bone morphogenic signaling [39]. Protein product binds bacterial LPS and Gram-negative bacteria, and has antibacterial function [40]. ***MIR186*** Expressed differentially in health gingiva versus periodontitis gingiva [41].
rs1003652	2	10,468,296	−3.563	0.734	4.39E-06	0.09572	0.4685	2.66E-05	***TAF1B*** Associated with non-syndromic cleft lip with palate in Chinese [42]. ***GRHL1*** Differentially expressed between gingiva and dental follicles [43]. Differentially regulated in primary pulp stem cells by enamel matrix derivative [44]. ***KLF11*** Involved in tooth development, specifically odontoblast differentiation [45]***.*** ***PDIA6*** Differentially expressed in soft tissue and bone after tooth extraction [46].
rs340349	2	179,933,412	4.79	0.833	2.20E-07	1.069	0.544	0.000184	
rs6434873	2	197,504,826	3.955	0.818	4.19E-06	−0.02973	0.3442	7.11E-06	***PGAP1*** Mouse gene knockout results in severe facial abnormalities, including lack of mouth, tongue, and mandible [47].
rs12488352	3	14,725,668	3.736	0.764	3.20E-06	−0.9612	0.526	4.07E-07	
rs6441084	3	156,310,776	4.57	0.904	3.26E-06				
chr3:194035416	3	194,035,416	−5.782	0.891	1.60E-07				***HES1*** Part of Notch signaling pathway involved in tooth development [48]. Promotes antimicrobial response in epithelial cells [49]. Regulates taste cell differentiation, specifically of the sweet-, bitter-, and umami-sensing cells [50]. ***LSG1*** Differentially expressed between the sexes in minor salivary glands [51].
rs62295581	4	11,844,859	6.403	1.173	2.05E-06	−0.6366	0.5008	**3.40E-08**	
chr4:158617368	4	158,617,368	7.46	1.375	2.76E-06				
rs28503910	4	182,680,709	4.983	0.973	4.84E-06	0.1327	0.4536	6.27E-06	***MIR1305*** Upregulated in smoker periodontal ligament-derived stem cells, and may impair the regenerative potential of this tissue [52].
rs12171500	5	76,460,134	15.92	2.507	1.37E-06	0.1616	0.4686	**6.46E-10**	***IQGAP2*** Involved in tooth development; upregulated in ameloblastoma [53]. ***S100Z*** Upregulated as part of ameloblastoma signature [54]. ***CRHBP*** One of most up-regulated genes in deciduous tooth pulp, as compared to that of permanent teeth [55]. ***F2R*** Encodes a transcription factor (PAR1) important for enamel formation [56].
rs12154393	7	11,210,931	3.828	0.762	3.06E-06	0.1274	0.3092	6.86E-06	***NDUFA4*** Candidate gene for role in cellular aging in dental follicle stem cells [57]. ***THSD7A*** Obesity candidate gene [58].
rs6947348	7	26,383,748	4.615	0.799	1.70E-07	0.3809	0.437	3.35E-06	***MIR148*** Involved in dental stem cells [59]. ***SNX10*** Mutations cause malignant osteopetrosis of infancy [60], a disease of increased bone mass that also has dental manifestations of delayed tooth eruption, congenitally missing or malformed teeth, and osteomyelitis and osteonecrosis of the jaw [61].
rs10224789	7	111,953,442	5.797	1.041	8.19E-07				***IFRD1*** Nociceptive pathway gene and risk factor for temporomandibular disorders [62].
rs817156	9	98,469,359	4.865	0.982	3.99E-06	−0.2586	0.3784	1.13E-06	***PTCH1*** Encodes the main receptor for the Hedgehog signaling pathway; mutations can cause odontogenic keratocysts, orofacial clefting, and hypodontia [63].
rs112246048	10	7,562,045	4.998	0.948	3.89E-06	−0.7608	0.6298	4.20E-07	
rs7107282	11	26,965,751	3.959	0.805	3.21E-06				***SLC5A12*** Lactate transporter in T-cells which enables T-cells to sense inflammatory environments [64]. ***CCDC34*** Bone mineral density-related candidate gene [65]. ***LGR4*** Required for sequential development of molars [66].
rs483743	13	113,786,947	3.82	0.743	1.39E-06	−0.08544	0.4209	4.82E-06	
rs4331298	15	71,918,351	4.054	0.818	3.01E-06	−0.6631	0.3067	**6.75E-08**	
rs72787939	16	26,556,887	4.617	0.819	6.34E-07	−1.076	0.4973	**2.81E-09**	
chr16:28719857	16	28,719,857	5.25	0.962	4.36E-06				***APOBR*** Associated with body fat percentage [67] (Lu et al., 2016). ***SH2B1*** Associated with BMI, and is implicated in leptin signaling [68].
rs2317828	16	82,266,591	3.72	0.727	1.55E-06				***PLCG2*** May play crucial role in odontogenesis [69]. ***CDH13*** Gene product may regulate the morphogenesis and rearrangement of secretory ameloblast cells [70].
rs321946	19	52,859,828	−3.552	0.716	2.54E-06	0.5165	0.4073	7.83E-07	
rs62225548	21	41,902,731	−3.693	0.700	6.41E-07	−0.2548	0.3871	1.71E-05	
rs2838538	21	45,687,271	4.606	0.914	4.34E-06	−0.1598	0.3027	7.48E-07	***CTSB*** Salivary levels of encoded protein are reduced in chronic graft-versus-host disease, which affects the saliva in the majority of patients [71]. ***AIRE*** Mutations cause autoimmune polyendocrinopathy candidiasis-ectodermal dystrophy, a feature of which can be dental abnormalities [72]. ***TRPM2*** Encodes an ion channel whose expression is increased in dental pulpitis. TRPM2 is activated in cancer radiation treatments to suppress Ca^2+^ signaling required for saliva production [73]. ***TSPEAR*** Mutations affect Notch signaling and cause an ectodermal dysplasia causing features including hypodontia [74].

Loci associated with caries, and genes within +/− 500 kb of the GWAS signal that have supporting evidence for a putative role in dental caries. Shown are lead SNPs of all loci meeting suggestive significance (*p*-value < 5 × 10^− 6^), their effect size in the Caucasian cohort and heterogeneity test p-value. Loci associated in the African-American cohort, but not found in the Caucasian GWAS don’t have values in the Caucasian cohort columns. Note: not all genes near GWAS signal are listed. *CHR* Chromosome, *BP* Basepair position. Bolded *p*-values are statistically significant (*p* ≤ 5 × 10^− 8^).

**Table 3:** Suggestive loci observed for DMFS

Lead SNP	CHR	BP	African-American cohort			Caucasian cohort		Q Statistic *P*-value	Nearby Gene(s) and Corroborating Evidence
			Effect Size	SE	*P*-value	Effect Size	SE		
rs1122613	1	3,768,565	−12.54	2.525	2.72E-06	1.395	1.121	4.56E-07	***ARHGEF16*** Candidate biomarker for diagnosis of oral squamous cell carcinoma [75].
rs1003652	2	10,468,296	−11.67	2.47	4.54E-06	1.04	1.497	9.86E-06	***TAF1B*** Candidate gene for susceptibility to non-syndromic cleft lip with palate [42]. ***GRHL1*** Differentially expressed between gingiva and dental follicles [43], differentially regulated in primary pulp stem cells by enamel matrix derivative [44]. ***KLF11*** Involved in tooth development, specifically odontoblast differentiation [45]. ***PDIA6*** Differentially expressed in soft tissue and bone after tooth extraction [46].
rs12488352	3	14,725,668	12.68	2.576	2.97E-06	− 3.552	1.679	1.30E-07	
chr3:194035416	3	194,035,416	−18.1	3.34	2.48E-06				***HES1*** Part of Notch signaling pathway involved in tooth development [48]. Promotes antimicrobial response in epithelial cells [49]. Regulates taste cell differentiation, specifically of the sweet-, bitter-, and umami-sensing cells [50]. ***LSG1*** Differentially expressed between the sexes in minor salivary glands [51].
rs28503910	4	182,680,709	17.13	3.055	1.99E-06	−0.9102	1.454	9.71E-08	
rs12171500	5	76,460,134	57.37	7.984	1.96E-06	0.8305	1.491	**3.37E-12**	***IQGAP2*** Involved in tooth development; upregulated in ameloblastoma [53]. ***S100Z*** Upregulated as part of ameloblastoma signature [54]. ***SNORA47*** Upregulated as part of ameloblastoma signature [54]. ***CRHBP*** One of most up-regulated genes in deciduous tooth pulp, as compared to that of permanent teeth [55]. ***F2R*** Encodes a transcription factor (PAR1) important for enamel formation [56].
rs6947348	7	26,383,748	14.59	2.698	1.38E-06	1.265	1.402	1.17E-05	***MIR148*** Involved in dental stem cells [59].
rs66691214	7	30,188,804	13.79	2.459	7.24E-07	0.9477	0.9689	1.18E-06	***FKBP14*** Mutations cause Ehlers-Danlos Syndrome [76], which can have oral manifestations [77]. ***NOD1*** Innate immunity gene expressed by dental pulp fibroblasts in the recognition of invaded caries-related bacteria and the subsequent innate immune responses [78]; gene product mediates sensing of periodontal pathogens [79], including *P. gingivalis* [80]. Required for the bone resorption consequences of immune activation by commensal bacteria in a model of periodontitis [81].
rs7011390	8	66,304,329	12.96	2.634	3.48E-06	0.7342	1.329	3.41E-05	
rs72787939	16	26,556,887	15.85	2.63	2.20E-07	−4.171	1.594	**7.51E-11**	***HS3ST4*** Heparan sulfate proteoglycans are coreceptors for FGFR22b, whose signaling is essential for progenitor survival and proliferation in several organs, including the submandibular gland and the tooth [82]
rs4801855	19	51,348,572	− 17.33	3.459	3.24E-06	− 0.4175	1.005	2.66E-06	***POLD1*** Mutations cause Mandibular Hypoplasia, Deafness and Progeroid features (MDP) syndrome, a premature aging syndrome which results in severe dental crowding and irregular teeth [83]. ***ACPT*** Recessive mutations in ACPT cause hypoplastic amelogenesis imperfecta; ACPT supplies phosphate during dentine formation [84]. ***KLK1*** Protein product is abundant in salivary proteome [85], and is involved in cellular inflammatory processes [86]. ***KLK4*** Homozygous mutations cause hypomaturation amelogenesis imperfecta [87]. ***SIGLEC9*** Modulates innate immunity [88]. ***CD33*** Encodes immunomodulatory receptor [89].

Loci associated with caries, and genes within +/− 500 kb of the GWAS signal that have supporting evidence for a putative role in dental caries. Shown are lead SNPs of all loci meeting suggestive significance (*p*-value < 5 × 10^− 6^), their effect size in the Caucasian cohort and heterogeneity test p-value. Loci associated in the African-American cohort, but not found in the Caucasian GWAS don’t have values in the Caucasian cohort columns. Note: not all genes near GWAS signal are listed. *CHR* Chromosome, *BP* Basepair position. Bolded *p*-values are statistically significant (*p* ≤ 5 × 10^− 8^).

### GWASs of caries in the primary dentition in African Americans

The dft GWAS yielded 46 suggestive SNPs across 17 distinct loci. The dfs GWAS yielded 32 suggestive SNPs across 17 distinct loci. Two loci overlapped between dfs and dft (rs2012033 and rs74574927/rs78777602). One notable suggestive locus, indicated by rs2515501 (*p*-value 4.54 × 10^− 6^), harbors antimicrobial peptide *DEFB1*. Gene annotations for the suggestive loci (p-value ≤5 × 10^− 6^) are listed in Table 4 (dft) and Table 5 (dfs). GREAT regulatory analysis results are available in the **Appendix.**

**Table 4:** Suggestive loci observed for dft

Lead SNP	CHR	BP	African-American cohort			Caucasian cohort		Q Statistic *P*-value	Nearby Gene(s) and Corroborating Evidence
			Effect Size	SE	*P*-value	Effect Size			
12,125,935	1	158,438,592	2.263	0.428	2.78E-06				***CD1D*** Gene product mediates mucosal immunity [90]. ***CD1A*** Encodes antigen-presenting protein expressed in oral epithelial cells [91]. ***CD1C*** Expressed in gingival environment on dendritic cells [92]. Locus contains clusters of ***OR6*** and ***OR10*** olfactory receptor family members [93]. ***PYHIN1*** Involved in inflammasome activation in host response to pathogens [94]. Asthma susceptibility locus specific to African-American ancestry [95].
rs11718323	3	20,915,514	9.527	1.462	2.37E-06	0.44	0.285	**1.06E-09**	
rs78777602	3	24,821,055	9.482	1.438	1.29E-06	−0.1403	0.2646	**4.67E-11**	***RARB*** Likely targeted by miRNAs involved in tooth morphogenesis and differentiation of dental cells [96]. Upregulated in ameloblastoma [53]. Has increased methylation in context of/is associated with head and neck squamous cell carcinoma, which is associated with dental hygiene and inflammation due to microbial factors [97].
rs1568206	3	106,639,719	3.087	0.505	9.33E-07	0.04032	0.2202	**3.25E-08**	
rs1505809	3	131,901,392	1.867	0.588	4.99E-06				
rs11932181	4	17,279,517	−2.104	0.410	2.18E-06				
rs1352733	4	112,280,742	2.372	0.443	3.47E-06	−0.1662	0.149	5.51E-08	
rs62316615	4	113,527,072	8.023	1.264	2.68E-06	−0.1937	0.2483	**1.79E-10**	GWAS signal is near gene desert that contains several enhancer elements that influence *PITX2* expression involved in Axenfeld-Rieger syndrome, which presents with dental anomalies (hypodontia, delayed primary tooth eruption, retrognathia of maxilla and mandible) [98]. ***LARP7*** Homozygous mutations cause Alazami syndrome, a feature of which is craniofacial dysmorphism [99]. ***MIR302A*** Promotes osteoblast differentiation [100].
rs57805404	4	190,403,652	3.667	0.608	1.54E-06				
rs11741099	5	178,664,463	1.96	0.400	2.93E-06	0.01215	0.1403	4.22E-06	***RUFY1*** Encodes protein that is part of the machinery that addresses periodontal pathogen intrusions in oral epithelial barriers [101]. ***MAML1*** Part of Notch signaling pathway, plays role in bone development [102]. ***GRM6*** Transcribed in a healthy gingival state [103]. GWAS signal is intronic variant to ***ADAMTS2***; homozygous mutations cause Ehlers-Danlos syndrome type VIIC, features of which include multiple tooth agenesis and dentin defects [104]. ADAMTS proteins are believed to play a role in various airway pathologies, including asthma [105].
rs61497549	7	5,842,842	2.343	0.448	2.78E-06				***MIR6874*** Upregulated in periodontal ligament cells when exposed to LPS of *P. gingivalis* [106].
rs1235058	7	54,614,145	2.256	0.425	3.14E-06	0.2763	0.1708	1.53E-05	***HPVC1*** Candidate gene for a complex chronic periodontitis trait involving a mixed infection bacterial community [107]. ***VSTM2A*** Exhibits high expression in mandibular molars relative to incisors [108]. ***EGFR*** EGF-receptors are found on the dental follicle, alceolar bone, and ameloblasts before and during tooth eruption [109, 110]. EGFR is a biomarker for neoplastic potential of dysplastic oral tissues [111]. Product mediates proliferation of gingival fibroblasts [112].
rs2515501	8	6,412,625	6.477	0.722	4.54E-06	−0.1906	0.1931	**4.39E-10**	***ANGPT2*** Upregulated in response to *P. gingivalis*; elevated levels are associated with oral squamous cell carcinoma [113]). ***DEFB1*** Encodes anti-microbial peptide; polymorphisms in *DEFB1* are associated with > 5 fold increase in DMFT/DMFS scores [114] and general DMFT index [115]. ***DEFB1, −A1, −A1B, −A1P, −A3, −A4, −A6, −A8P, −9P, −A10, −T1P2*** The defensin family (**DEF**-) of antimicrobial peptides (Ganz 2003), is involved in chronic periodontal inflammation [116], and oral carcinogenesis [117].
rs10504504	8	72,185,924	4.625	0.771	3.23E-06	−0.06708	0.2221	**4.98E-09**	
rs74949229	10	9,526,946	7.142	0.455	4.38E-06	0.2898	0.2229	**2.53E-09**	
rs6585998	10	87,943,609	− 2.107	0.428	2.78E-06	−0.18	0.1382	1.79E-05	***MIR346*** Involved in osteogenic differentiation of human bone marrow mesenchymal stem cells [118].
rs11020123	11	92,689,278	−2.269	0.991	4.10E-06	0.13	0.1328	4.16E-07	***FAT3*** One of genes enriched in the GO category “calcium ion binding” that are differentially expressed throughout different stages of tooth development [119]. ***MTNR1B*** Locus associated with type II diabetes [120].
rs79812076	12	102,989,156	6.012	0.967	2.17E-06	0.5207	0.2254	**3.16E-08**	***IGF1*** Encodes metabolism regulator of hard dental tissue through action on IGF1-receptor; is involved in later stages of tooth development and pulpal differentiation [121].
rs9630337	13	68,582,970	6.97	1.128	4.47E-06	−0.5214	0.2149	**3.07E-11**	
chr13:96271864	13	96,271,864	8.184	1.308	3.62E-06				***CLDN10*** Expressed in preodontoblasts, and restricted to the lingual basal epithelium of the tooth bud [122]. ***HS6ST3*** Associated with obesity (BMI ≥ 35 kg/m2) [123].
rs422342	15	67,347,686	2.865	0.543	2.39E-06	0.31	0.2138	1.20E-05	***SMAD6*** Highly expressed in bone, including mandible and palatal bone [124]. ***SMAD3*** Expressed in intramembranous bones and submandibular salivary gland [124]. ***MAP 2 K5*** Associated with BMI [125].
rs7174369	15	99,830,322	2.586	0.482	1.72E-06	0.1903	0.1358	1.68E-06	***MEF2A*** Regulates osteogenic differentiation of dental pulp stem cells [126]. ***IGF1R*** IGF-receptor; involved in signaling in dental fibroblast apoptosis [127].
rs9915753	17	73,011,448	3.814	1.107	4.50E-06	−0.02813	0.143	1.81E-07	***SLC9A3R1*** Mutations cause hypophosphatemic nephrolithiasis/osteoporosis 2, features of which can include skeletal defects, fractures, and osteoporosis/osteopenia [128].
rs2012033	19	34,163,298	2.248	0.424	8.21E-07	0.1823	0.1637	5.37E-06	***CHST8*** Candidate gene for hypodontia [129]. ***KCTD15*** Associated with obesity and preference for carbohydrates [130].
rs55928325	19	56,507,948	3.22	1.051	4.70E-06	0.06884	0.2314	6.11E-07	***NLRP5*** Increased expression in mucosa in state of periodontitis [131], and part of gene expression network preferentially expressed in lower incisors [108]. ***ZNF582*** Hypermethylation is associated with oral dysplasia and cancer [132].

Loci associated with caries, and genes within +/− 500 kb of the GWAS signal that have supporting evidence for a putative role in dental caries. Shown are lead SNPs of all loci meeting suggestive significance (*p*-value < 5 × 10^− 6^), their effect size in the Caucasian cohort and heterogeneity test p-value. Loci associated in the African-American cohort, but not found in the Caucasian GWAS don’t have values in the Caucasian cohort columns. Note: not all genes near GWAS signal are listed. *CHR* Chromosome, *BP* Basepair position. Bolded *p*-values are statistically significant (*p* ≤ 5 × 10^− 8^).

**Table 5:** Suggestive loci observed for dfs

Lead SNP	CHR	BP	African-American cohort			Caucasian cohort		Q Statistic *P*-value	Nearby Gene(s) and Corroborating Evidence
			Effect Size	SE	P-value	Effect Size			
rs11240576	1	205,816,923	6.548	1.338	4.36E-06				
rs74574927	3	24,768,865	16.13	2.251	2.44E-06	−0.6646	0.6546	**7.83E-13**	***RARB*** Likely targeted by miRNAs involved in tooth morphogenesis and differentiation of dental cells [96]. Upregulated in ameloblastoma [53]. Has increased methylation in context of/is associated with head and neck squamous cell carcinoma, which, in turn, is associated with dental hygiene and inflammation due to microbial factors [97].
rs7630386	3	29,024,237	22.38	2.805	9.51E-07				***RBMS3*** Candidate gene for a complex chronic periodontitis trait involving a periodontal pathogen load [107].
rs1505809	3	131,901,392	5.215	1.089	9,301,573				
rs552922	3	150,281,061	6.1	1.074	4.19E-06	0.08645	0.2895	6.44E-08	
rs11932181	4	17,279,517	−5.766	1.66	4.94E-06				
rs1602815	4	131,105,072	7.756	1.35	2.47E-06	0.1524	0.3582	5.21E-08	
rs36162355	5	2,246,469	11.62	1.926	4.38E-06				***IRX4*** Differentially expressed in incisors versus molars [108]. ***IRX2*** Localizes to cervical loop during replacement tooth morphogenesis in fish model [133].
rs17606253	6	111,526,445	12.19	1.928	1.85E-06	0.2342	0.4277	**1.41E-09**	***TRAF3IP2*** Involved in mucosal immunity and IL-17 signaling, and associated with a complex chronic periodontitis trait involving high levels of A. *actinomycetemcomitans* and a profile of aggressive periodontal disease [107].
rs10815750	9	8,087,319	5.522	1.05	1.55E-06	0.1317	0.3258	9.44E-07	
rs1434274	9	8,817,244	6.899	1.306	4.53E-06	−0.08817	0.3447	2.30E-07	
rs11008779	10	32,413,987	9.853	1.725	3.22E-06	−0.4776	0.3681	**4.72E-09**	
rs6585998	10	87,943,609	−5.642	1.184	4.45E-06	−0.5171	0.3351	3.12E-05	***MIR346*** Predicted to regulate a gene related to calcium binding during amelogenesis [119].
rs6483205	11	92,669,908	−7.844	1.396	1.24E-06	0.4095	0.3187	**8.22E-09**	***FAT3*** Gene enriched in the GO category “calcium ion binding” and are differentially expressed throughout different stages of tooth development [119]. ***MTNR1B*** Polymorphisms associated with fasting glucose [134] and type 2 diabetes [135].
rs61950818	13	63,216,679	15.35	2.346	2.70E-06	− 0.01181	0.4964	**1.49E-10**	
rs9915753	17	73,011,448	12.24	1.905	3.60E-07	−0.1695	0.348	**1.47E-10**	***CD300E*** Significantly upregulated in healing gingiva [136]. ***SLC9A3R1*** Heterozygous mutations cause hypophosphatemic nephrolithiasis/osteoporosis which causes decreased bone mineral density [128].
rs2012033	19	34,163,298	5.763	1.136	1.40E-06	0.6308	0.3967	2.00E-05	***CHST8*** Candidate gene for hypodontia [129]. ***KCTD15*** Associated with obesity and preference for carbohydrates [130].

Loci associated with caries, and genes within +/− 500 kb of the GWAS signal that have supporting evidence for a putative role in dental caries. Shown are lead SNPs of all loci meeting suggestive significance (*p*-value < 5 × 10^− 6^), their effect size in the Caucasian cohort and heterogeneity test p-value. Loci associated in the African-American cohort, but not found in the Caucasian GWAS don’t have values in the Caucasian cohort columns. Note: not all genes near GWAS signal are listed. *CHR* Chromosome, *BP* Basepair position. Bolded *p*-values are statistically significant (*p* ≤ 5 × 10^− 8^).

### Comparison with Caucasian caries GWAS

Results of the tests for heterogeneity between African Americans and Caucasians are listed in Table 6. Significant (p-value ≤5 × 10^− 8^) heterogeneity in effects between racial groups was observed for 50% of the loci in children, and 12–18% of loci in adults.

**Table 6:** Loci showing significant heterogeneity between African Americans and Caucasians caries GWASs

Phenotype	SNP	CHR	BP	P-value (AA)	Effect Size (AA)	SE (AA)	Effect Size (C)	SE (C)	Q Statistic *P*-value
DMFT	rs62295581	4	11,844,859	2.05E-06	6.403	1.173	− 0.6366	0.5008	3.40E-08
	rs12171500	5	76,460,134	1.37E-06	15.92	2.507	0.1616	0.4686	6.46E-10
	rs4331298	15	71,918,351	3.01E-06	4.054	0.818	−0.6631	0.3067	6.75E-08
	rs72787939	16	26,556,887	6.34E-07	4.617	0.8189	−1.076	0.4973	2.81E-09
DMFS	rs12171500	5	76,460,134	1.96E-06	57.37	7.984	0.8305	1.491	3.37E-12
	rs72787939	16	26,556,887	2.20E-07	15.85	2.63	−4.171	1.594	7.51E-11
dft	rs11718323	3	20,915,514	2.37E-06	9.527	1.462	0.44	0.285	1.06E-09
	rs78777602	3	24,821,055	1.29E-06	9.482	1.438	−0.1403	0.2646	4.67E-11
	rs1568206	3	1.07E+ 08	9.33E-07	3.087	0.5053	0.04032	0.2202	3.25E-08
	rs1112769	3	1.88E+ 08	3.65E-06	5.947	0.9909	−0.3341	0.2187	6.02E-10
	rs62316615	4	1.14E+ 08	2.68E-06	8.023	1.264	−0.1937	0.2483	1.79E-10
	rs2515501	8	6,412,625	4.54E-06	6.477	1.051	−0.1906	0.1931	4.39E-10
	rs10504504	8	72,185,924	3.23E-06	4.625	0.771	−0.06708	0.2221	4.98E-09
	rs74949229	10	9,526,946	4.38E-06	7.142	1.128	0.2898	0.2229	2.53E-09
	rs79812076	12	1.03E+ 08	2.17E-06	6.012	0.9667	0.5207	0.2254	3.16E-08
	rs9630337	13	68,582,970	4.47E-06	6.97	1.107	−0.5214	0.2149	3.07E-11
dfs	rs74574927	3	24,768,865	2.44E-06	16.13	2.251	−0.6646	0.6546	7.83E-13
	rs17606253	6	1.12E+ 08	1.85E-06	12.19	1.928	0.2342	0.4277	1.41E-09
	rs11008779	10	32,413,987	3.22E-06	9.853	1.725	−0.4776	0.3681	4.72E-09
	rs61950818	13	63,216,679	2.70E-06	15.35	2.346	−0.01181	0.4964	1.49E-10
	rs9915753	17	73,011,448	3.60E-07	12.24	1.905	−0.1695	0.348	1.47E-10
	rs74574927	3	24,768,865	2.44E-06	16.13	2.251	−0.6646	0.6546	7.83E-13

*CHR* Chromosome, *BP* Basepair, *AA* African American, *C* Caucasian. Significance threshold is *p*-value ≤5 × 10–^8^.

## Discussion

Dental caries is a complex disease that disproportionately affects certain groups, including African Americans.

This is one of few studies of the genetics of dental caries to specifically investigate African Americans. The purpose of this pilot study was to perform preliminary GWAS scans in African American children and adults and to contrast the evidence for genetic association between Africans Americans and Caucasians.

Though no significant associations were observed (which was expected given the small samples sizes), several suggestive loci showed strong evidence of genetic heterogeneity between African Americans and Caucasians. These findings suggest that the genetic architecture of dental caries differs across racial groups. Thus, gene-mapping efforts in African American and other minority racial groups are warranted, and may lead to the discovery of caries risk loci that would go undetected by studying Caucasians alone.

Several suggestive loci harboring genes with putative connections to caries were observed. Given the exploratory nature of this study, we describe suggestive hits to potentially help inform new hypotheses about caries genetics. We caution that these suggestive loci should be interpreted with much skepticism.

### GWASs of permanent dentition in African Americans

Several themes emerged from annotation of suggestively associated genes, including saliva-, salivary gland-, and salivary proteome-related genes. A gene encoding a salivary protein involved in inflammatory processes (*KLK1*; rs4801855; *p*-value 3.24 × 10^− 6^) [85, 86], a transcription factor differentially expressed in the minor salivary glands between the sexes (*LSG1*; chr3:194035416; *p*-value 1.6 × 10^− 7^) [51], and a gene encoding a salivary protein (*CTSB*; rs2838538; p-value 4.34 × 10^− 6^) were identified.

Several genes related to the immune response and periodontal disease were identified. *HES1 (*chr3:194035416) encodes a transcription factor with roles in antimicrobial response within epithelial cells [49]. *NOD1* (rs66691214; *p*-value 7.24 × 10^− 7^) encodes a dental pulp protein with roles in sensing caries-related [78] and periodontal pathogens [79, 80], and the subsequent immune response [78, 81]. Protein products of several genes are involved in innate immunity [64, 88] (*SIGLEC9, CD33;* rs4801855; *p*-value 3.24 × 10^− 6^ and *SLC5A12;* rs7107282; *p*-value 3.21 × 10^− 6^). *PTGER3* (rs74086974; p-value 3.18 × 10^− 6^) is a candidate gene for the outcome of periodontal disease therapy [38], and *MIR186* (rs74086974) is differentially expressed between gingiva in health versus periodontitis [41]. rs28503910 (*p*-value 4.84 × 10^− 6^) contained *MIR1305,* which is upregulated in response to smoking and may impair regeneration of periodontal tissues in that state [52]. *TRPM2* (rs2838538; *p*-value 4.34 × 10^− 6^) encodes an ion channel upregulated in dental pulpitis [137], and is involved in saliva production [138].

Tooth and enamel development-related genes were present across several loci, including a gene associated at nominal significance, *TUFT1* (rs11805632; *p*-value 5.15 × 10^− 6^), which had previously been found to be associated with dental caries in Caucasian children and adults, and which displays interaction with fluoride exposure [8]. Additional genes included *HS3ST4* (rs72787939; *p*-value 2.20 × 10^− 7^), which encodes a co-receptor essential for submandibular gland and tooth progenitor function [82]. Genes with roles in dental stem cells (*MIR148A*; rs6947348; p-value 1.38 × 10^− 6^) [59], and a locus with genes involved in tooth development (*IQGAP2*; rs12171500; p-value 1.96 × 10^− 6^) [53], enamel formation (*F2R*) [56], deciduous tooth pulp (*CRHBP*) [55], and ameloblastoma (*S100Z, SNORA47, IQGAP2*) [53, 54], were found. Also, previously-mentioned *HES1* (chr3:194035416) has a role in tooth development [48], and taste cell differentiation [50]. The rs2317828 locus (*p*-value 1.55 × 10^− 6^) contains genes that play a crucial role in odontogenesis (*PLCG2)* [56] and ameloblast development (*CDH13*) [70]. *LGR4* (rs7107282; p-value 3.21 × 10–6) is required for the sequential development of molars [66]. *FOXF2* (rs2814820; *p*-value 3.90 × 10^− 6^) and *TAF1B* (rs1003652; p-value 4.54 × 10^− 6^) are near a cleft lip [139] and cleft lip and palate risk loci [88], respectively. *FOXF2* also encodes a protein located near tooth germ cells during tooth development [140]. The rs1003652 (p-value 4.54 × 10^− 6^) locus includes several genes that are differentially expressed between various dental, bone, or gingival tissues (*GRHL1, PDIA6*) [44, 46], and one involved in odontoblast development (*KLF11*) [45].

Finally, several genes are involved in monogenic disorders with dental phenotypes, including S*NX10* (malignant osteopetrosis of infancy, which can have features of delayed tooth eruption, missing or malformed teeth; rs6947348; *p*-value 1.7 × 10^− 7^) [61], a locus containing *POLD1* (mandibular hypoplasia, deafness, progeroid features; rs4801855; 3.24 × 10^− 6^) [83], *ACPT* (hypoplastic amelogenesis imperfecta) [84], *KLK4* (hypomaturation amelogenesis imperfecta) [87], a locus containing *AIRE* (autoimmune polyendocrinopathy candidiasis-ectodermal dystrophy, which can feature dental abnormalities; rs2838538; *p*-value 4.34 × 10^− 6^) [72], and *TSPEAR* (ectodermal dysplasia causing hypodontia) [74].

The locus chr16:28719857 (*p*-value 4.36 × 10^− 6^) contains genes associated with body fat percentage (*APOBR*) [67] and BMI (*SH2B1*) [68], and rs12154393 (p-value 3.06 × 10–6) contains *THSD7A*, a candidate gene for obesity [58].

### GWASs of primary dentition in African Americans

The locus near rs2012033 was associated in both primary caries GWASs (dft *p*-value 8.21 × 10^− 7^; dfs p-value 1.40 × 10^− 6^) and harbored a candidate gene for hypodontia (*CHST8*) [129] and a gene associated with obesity and preference for carbohydrate (*KCTD15*) [130]. Other loci with connections to obesity and related disorders include chr13:96271864 (*p*-value 3.62 × 10^− 6^) that harbors the obesity-associated gene *HS6ST3* [123], rs422342 (2.39 × 10^− 6^), which includes *MAP 2 K5*, also associated with BMI [125], and rs6483205 (p-value 1.24 × 10^− 6^) which contains *MTNR1B*, polymorphisms in which are associated with fasting glucose [134] and type 2 diabetes [135].

The locus rs2515501 (p-value 4.54 × 10^− 6^) harbored several members of the alpha and beta defensin family of antimicrobial peptides [141], which are involved in chronic periodontal inflammation [116] and oral carcinogenesis [117]. Of note, this locus contains *DEFB1*, polymorphisms in which are associated with a > 5 fold increase in DMFT and DMFS scores [114], and general DMFT index [115]. An additional gene at this locus, *ANGPT2*, is also associated with oral cancer, and upregulated in response to *P. gingivalis,* a periodontal pathogen [113].

Three separate associated loci harbored genes associated with complex periodontal traits, proxies for different subgroups of periodontal disease, a condition closely associated with dental caries [142]. rs1235058 (*p*-value 3.14 × 10–6) harbored *HPVC1*, a candidate gene for a trait involving a mixed infection bacterial community [107]. rs7630386 (p-value 9.51 × 10^− 7^) harbored *RBMS3*, a candidate gene for a trait involving a high periodontal pathogen load [107]. Thirdly, rs17606253 (*p*-value 1.85 × 10^− 6^) harbored *TRAF3IP2*, a protein implicated in mucosal immunity and IL-17 signaling, and associated with a trait involving high levels of *A. actinomycetemcomitans* and a profile of aggressive periodontal disease [107].

Two loci were found to be related to asthma, a disease associated with a doubled risk of caries [143]. rs12125935 (*p*-value 2.78 × 10^− 6^) harbors *PYHIN1*, which encodes a protein involved in inflammasome activation in response to pathogens [94], and represents an asthma susceptibility locus specific to African-American ancestry [95]. rs11741099 (*p*-value 2.93 × 10^− 6^) is intronic to *ADAMTS2*; the ADAMTS protein family is proposed to play a role in asthma [105]. Additionally, homozygous mutations in *ADAMTS2* cause Ehlers-Danlos syndrome (VIIC), features of which can include multiple tooth agenesis and dentin defects [104].

rs7174369 (*p*-value 1.72 × 10^− 6^) harbored *IGF1R*, involved in dental fibroblast apoptosis [127]. Interestingly, in addition to its receptor, the regulator of hard dental tissue encoded by *IGF1* was also associated at a separate locus (rs79812076; p-value 2.17 × 10^− 6^).

### Comparison between association results across dentition type and across races

Aside from *TUFT1* and *DEFB1,* the loci reported here have not been associated with dental caries in previous studies, which have largely comprised Caucasian individuals. This is in line with previous research showing differences in frequencies of risk alleles for complex disease across races, but may also be because the study was underpowered to detect associated loci in African Americans. In addition, no overlap was found in associated loci between this study and a multi-ethnic pilot GWAS of early childhood caries [144]. There was no overlap in loci associated with primary and permanent caries indices, but this might be expected given that the genetic determinants of caries are thought to largely differ between the dentitions [6]. However we cannot rule out similarities in genetic determinants across dentitions because this pilot study was not designed to have sufficient power for this purpose.

Loci displaying significant heterogeneity between African Americans and Caucasians (Table 6) in permanent dentition were largely ones in gene deserts with unknown function. One locus (rs12171500; DMFT Q statistic [Q] *p*-value 6.46x^− 10^; DMFS Q *p*-value 3.37x^− 12^) contained genes involved in enamel and tooth development.

Among loci displaying significant heterogeneity in primary dentition, there were several that harbored genes related to periodontitis. Such loci represented genes related to periodontal inflammation (rs2515501; Q p-value 4.39x^− 10^), gingival healing (rs9915753; dft Q p-value 1.81x^− 07^, dfs Q p-value 1.47x^− 10^), and aggressive periodontal disease and high levels of oral *A. actinomycetemcomitans* (rs17606253; Q p-value 1.41x^− 9^). Notably, African American pre-teens are approximately 16 times as likely as Caucasian ones to have localized aggressive periodontitis and detection of *A. actinomycetemcomitans* is associated with early surrogates for periodontal inflammation in African American preadolescents [145].

Several broad categories of genes associated with caries in African Americans emerged, including those involved in tooth/enamel development, those causing single-gene disorders with craniofacial or dental malformations, those involved in immune response or periodontitis, those related to salivary glands and proteins, and those associated with obesity. These results support the known multifactorial nature of dental caries [21]. Further studies will be necessary to confirm the loci nominated in this pilot study. Nevertheless, these GWASs provide valuable insight into the differences in the genetic architecture of caries across populations, and suggest new candidate genes worth following-up in hypothesis-driven studies.

### Study limitations

This study has limitations, including the genotyping platform, which was not optimized for genomic coverage of the African American population [146, 147]. Thus, studies in larger African American cohorts and with denser chips are needed to identify risk loci that may not have been well represented in this study. The ascertainment of caries was limited by the lack of X-ray examination to confirm white spots and approximal tooth surface caries, which would have underestimated the true extent of caries counts. Imprecision in the caries assessment would lower the power to detect association, but would not result in false positive associations. Therefore, the associations observed in this study would likely not be influenced by this limitation, but other true associations may have gone undetected. The pediatric cohort analyses were somewhat limited in that the primary caries indices (dfs/dft) were tested for genetic association in a sample that included some children with mixed dentition. Limiting the scope of the pediatric analyses to solely primary dentition caries indices allows for simplified interpretation of the association results because genetic determinants of primary and permanent tooth caries have been found to differ [6]. However, assessing dfs/dft scores in the mixed dentition provides an incomplete picture of the caries experience in the primary dentition, given the exfoliation of some teeth. This is another important source of measurement error, which would bias our analysis toward the null hypothesis of no association.

## Conclusions

In summary, these results suggest that there may be genetic differences in caries susceptibility, and potentially differing genetic etiologies or differentially distributed genetic risk factors, across racial groups. Indeed, addressing the oral health disparity gap is a national priority according to both the US Surgeon General’s Oral Health in America report [12] and the Healthy People 2020 public health goal framework [148]. This oral health disparity has parallels in the research sphere - relatively little work, to date, has been done on the genetics of caries in African Americans. Furthermore, African Americans represent a segment of the population traditionally underrepresented in biomedical research (UBR) and the importance of including such groups in research is recognized as foundational to the future of precision medicine by the National Institutes of Health initiative, All of Us [149]. Larger gene-mapping studies are thus needed in this population to help alleviate its disproportionate burden of the disease.

## Data Availability

The datasets analysed during the current study are available in the dbGaP repository (Study Accession: phs000095.v3.p1). Senior and NIH Investigators are eligible to apply for access.

## Abbreviations

dfs: Number of decayed or filled primary tooth surfaces
dft: Number of decayed or filled primary teeth
DMFS: Number of decayed, missing, or filled permanent tooth surfaces
DMFT: Number of decayed, missing, or filled permanent teeth
GWAS: Genome-wide association study
MAF: Minor allele frequency
SNP: Single nucleotide polymorphism
